# Anchoring the T6SS to the cell wall: Crystal structure of the peptidoglycan binding domain of the TagL accessory protein

**DOI:** 10.1371/journal.pone.0254232

**Published:** 2021-07-02

**Authors:** Van Son Nguyen, Silvia Spinelli, Éric Cascales, Alain Roussel, Christian Cambillau, Philippe Leone

**Affiliations:** 1 Architecture et Fonction des Macromolécules Biologiques, Aix-Marseille Université, Marseille, France; 2 Architecture et Fonction des Macromolécules Biologiques, Centre National de la Recherche Scientifique (UMR7257), Marseille, France; 3 Laboratoire d’Ingénierie des Systèmes Macromoléculaires, Institut de Microbiologie de la Méditerranée, Centre National de la Recherche Scientifique (UMR7255), Aix-Marseille Université, Marseille, France; Academia Sinica, TAIWAN

## Abstract

The type VI secretion system (T6SS) is a widespread mechanism of protein delivery into target cells, present in more than a quarter of all sequenced Gram-negative bacteria. The T6SS constitutes an important virulence factor, as it is responsible for targeting effectors in both prokaryotic and eukaryotic cells. The T6SS comprises a tail structure tethered to the cell envelope via a trans-envelope complex. In most T6SS, the membrane complex is anchored to the cell wall by the TagL accessory protein. In this study, we report the first crystal structure of a peptidoglycan-binding domain of TagL. The fold is conserved with members of the OmpA/Pal/MotB family, and more importantly, the peptidoglycan binding site is conserved. This structure further exemplifies how proteins involved in anchoring to the cell wall for different cellular functions rely on an interaction network with peptidoglycan strictly conserved.

## Introduction

During evolution, Gram-negative bacteria have evolved sophisticated mechanisms to hunt their prey or run away from their predators. Some of the most important examples are bacterial secretion systems, specialized nano-machines utilized by bacteria to transport virulence factors across the cell envelope [1,2]. The type VI secretion system is a widespread mechanism found in more than a quarter of all sequenced Gram-negative bacteria, with one or several copies in each species [3–5]. This nanomachine transports protein effectors directly from the cytoplasm across the cell envelope and injects them into the target cells, both prokaryotic and eukaryotic [6–9]. In pathogenic bacteria, the T6SS has been shown to participate in virulence processes, such as the translocation of toxin proteins into eukaryotic cells, leading to cytoskeleton rearrangement, cell rounding and death or cytotoxicity [10–13]. The T6SS has also been shown to be involved in stress sensing, cell mobility and biofilm formation [14–16]. The T6SS is therefore considered a versatile weapon that meets the need of each T6SS^+^ bacterial species [17].

The T6SS functions by contraction of the TssBC sheath to force the spike-tube structure out of the cell, puncturing the target cell and delivering toxin effectors [6,18–20]. The nano-machine is composed of at least thirteen core-components [3,4] named TssA to TssM, (“Tss” named from “Type six secretion system” [21]). Two hallmark proteins, Hcp (haemolysin-coregulated-protein) and VgrG (valine-glycine repeated protein), are found in the culture supernatant of T6SS^+^ bacteria [3]. The tail sheath polymerizes from a baseplate consisting of at least four proteins: TssK, TssF, TssG, and TssE [22–24].

The baseplate-sheath structure is anchored to the cell trans-envelope via a membrane-associated complex [25–27]. This complex comprises three T6SS core components: the TssL and TssM inner membrane proteins and the TssJ outer membrane lipoprotein [26,28–31], presumably making a trans-periplasmic channel for passage of the tail tube/spike [27]. Proper assembly of the TssJLM complex requires the local degradation of the cell wall, which is accomplished by the recruitment of a peptidoglycan (PG)-degrading enzyme [32,33]. It has been proposed that the TssJLM complex undergoes a large conformational change during the injection process [27]. The TssJLM complex should be stably anchored to the cell envelope to ensure its maintenance when the sheath contracts. Interestingly, with the notable exceptions of *Vibrio cholerae* and *Edwardsiella tarda*, there is a peptidoglycan-binding domain (PGBD) that anchors the T6SS to the cell wall [6,25,26]. Other secretion systems spanning both the outer and inner membranes comprise also a component harbouring a PGDB. ExA from the *Aeromona hydrophila* T2SS was shown to cross-link with PG [34], and recently, the structure of the PorE PGBD from the *Porphyromonas gingivalis* T9SS was solved in complex with a PG fragment [35]. In many T6SSs, the PGBD is fused to the C terminus of TssL, which are then called “specialized TssL”. In some other T6SSs, the PGBD is carried by accessory T6SS subunits, such as TagL, TagP, TagN and TagW [25,26]. These accessory proteins are usually encoded within T6SS clusters. Although no data is available for TagP, TagN and TagW, the entero-aggregative *Escherichia coli* (EAEC) TagL subunit has been studied for its PG-binding function [25,26]. TagL interacts with the TssJLM membrane complex through a direct interaction with TssL [25]. A topology study has defined that TagL comprises two trans-membrane segments at the N terminus, followed by a cytoplasmic domain and a third trans-membrane segment before the periplasmic domain. The TagL periplasmic domain includes a PGBD of the OmpA/Pal/MotB family (PF00691) at its C terminus, located between residues 442 and 556. This domain binds *in vitro* and *in vivo* to the PG layer, and mutations within the PG-binding motif abolish both the interaction with the cell wall and the function of the T6SS [25].

Although binding to the cell wall is important for T6SS assembly and function, no structure is available for any of these domains. In this study, we solved the structure of the PGBD of the EAEC TagL protein. The structure is similar to those of known PGBD of the OmpA/Pal/MotB family, and the PG binding pocket is conserved, suggesting that T6SS anchoring to PG is mainly mediated by interactions with the diaminopimelic acid (DAP) residue.

## Materials & methods

### Production of TagL Peptidoglycan-binding domain (TagL PGBD)

A DNA fragment corresponding to residues 440–552 of the TagL PGBD from the EAEC strain 17–2 was cloned into the pDEST17 expression vector using the Polymerase Incomplete Primer Extension (PIPE) cloning method [36]. The final construct encodes the TagL PGBD fused to a N terminal hexahistidine tag followed by a TEV cleavage site. This construct was then produced and purified as described previously [29]. Briefly, the expression vector was transformed into *E*. *coli* T7 pLysS cells (New England Biolabs). Cells were grown in Terrific Broth (TB) at 37°C until the optical density at 600 nm reached 0.6–0.8. Expression of the TagL PGBD was induced by 0.5 mM isopropyl-β-thio-galactoside (IPTG) for 16 hours at 17°C. Cells were harvested at 4,000 × *g* for 15 minutes at 4°C, resuspended in lysis buffer (50 mM Tris-HCl, pH 8, 300 mM NaCl, 10 mM imidazole, 0.25 mg/mL lysozyme) and lysed by sonication. The lysate was cleared by centrifugation at 18,000 × *g* for 45 minutes and the supernatant loaded onto a Ni^2+^ affinity chromatography column (HisTrap 5 mL, GE Healthcare). The protein was eluted with a step gradient of 250 mM imidazole. The hexahistidine tag was cleaved by incubation of the protein with the histidine-tagged TEV protease (ratio 10:1, protein:TEV, w/w) overnight, coupled with dialysis to eliminate imidazole. The untagged TagL PGBD was obtained in the flow through of a second Ni^2+^ affinity chromatography and further purified by gel filtration on a preparative Superdex 75 column (GE Healthcare) equilibrated in 20 mM Tris-HCl, pH 8, 150 mM NaCl. The TagL PGBD was concentrated to 5 mg/mL for crystallization.

### Crystallization and data processing of TagL PGBD

Crystallization trials were carried out using the sitting-drop vapour diffusion method at 20°C in 96-well Greiner crystallization plates. The reservoirs of the Greiner plates were filled with a TECAN pipetting robot, and nanodrops were dispensed by a Mosquito robot (TTP Labtech). Crystals of TagL PGBD appeared after 1 day by mixing a protein solution (5 mg/mL) with 0.1 M imidazole, pH 6.5, and 1.2 M sodium acetate. Crystals were cryo-cooled in a well solution supplemented with 25% glycerol in a flow of liquid nitrogen. Datasets were collected at the European Synchrotron Radiation facility (ESRF, Grenoble, France), and were processed using the XDS package [37]. TagL PGBD crystals belonged to space group P6_3_, a = b = 75.9 Å, c = 177.7 Å; α = β = 90°, γ = 120°, with four molecules per asymmetric unit. The structure of TagL PGBD was solved by molecular replacement using MolRep [38] with the OmpA-like domain from *A*. *baumannii* (3TD3) as initial model. The structure was corrected and refined with COOT [39] and BUSTER [40], respectively. The validity of the refined structure was assessed by MolProbity [41]. Data collection and refinement statistics are given in Table 1; the atomic coordinates and structure factors have been deposited at the Protein Data Bank (PDB) under accession code 7BBA.

**Table 1:** Data collection and refinement statistics of the TagL peptidoglycan binding domain (PGBD).

**Data collection**	
Space group	P6_3_
*a*, *b*, *c* (Å)	75.9, 75.9, 177.6
α, β, γ (°)	90, 90, 120
Number of monomers	4
Resolution limits (Å)*	34.9–2.43 (2.57–2.43)
R_merge_*	0.125 (0.90)
CC1/2	0.991 (0.777)
Unique reflections*	21834 (3454)
I/σ*	11.7 (1.8)
Completeness (%)*	99.7 (98.2)
Multiplicity*	21.1 (22)
**Refinement**	
Resolution (Å)*	34.9–2.43 (2.45–2.43)
Reflections*	21796 (436)
R_work_/R_free_ (%)	23.8/24.6
Number of atoms: protein/water/ion	3463/176/45
Rmsd.: bond (Å)/angles (°)	0.009/1.07
B-factors (Å^2^): protein/water/ion	76.0/64.0/87.3
Ramachandran (%): preferred/allowed/outliers	91.8/7.3/0.9
PDB accession code	7BBA

* Values in parentheses are for the highest-resolution shell.

## Results

### Sequence analysis and TagL PGBD purification

**Fig 1:**
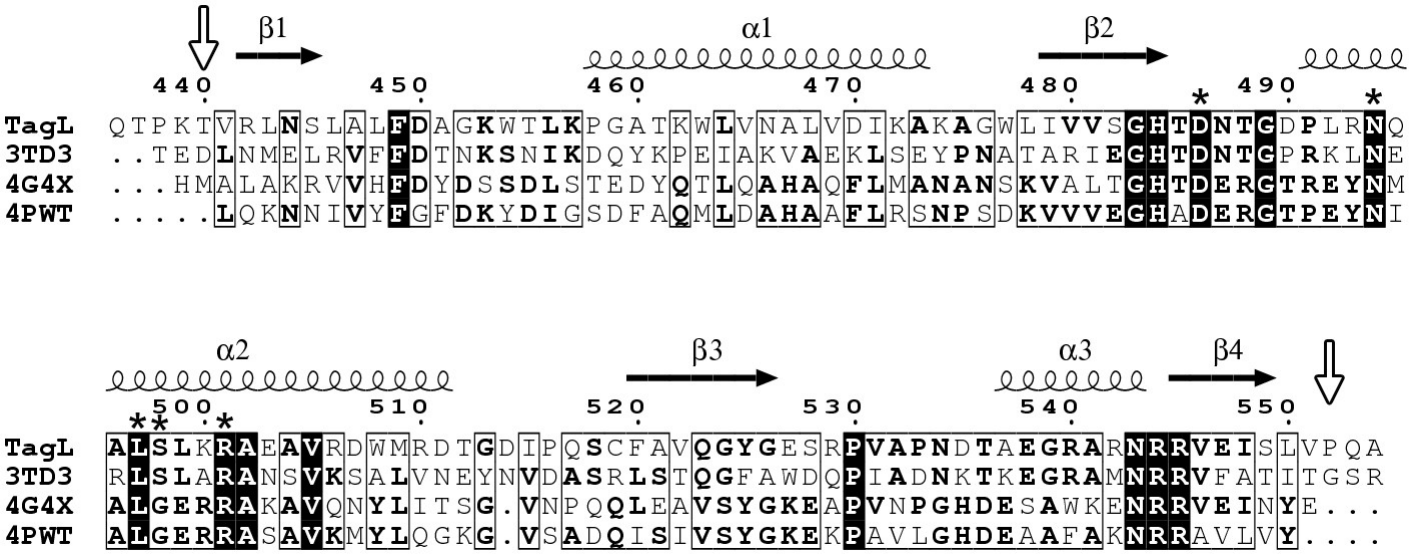
Sequence alignment of the TagL peptidoglycan binding domain (PGBD) with other representative OmpA family proteins. Secondary structure elements and numeration from the TagL PGBD structure are displayed above the alignment. Strictly conserved residues are in black blocks; the five residues that were shown to be required for TagL binding to the cell wall are indicated by an asterisk; selected N and C termini of the TagL PGBD are indicated by vertical arrows. 3TD3: OmpA from *Acinetobacter baumannii*; 4G4X: Pal from *Acinetobacter baumannii*; 4PWT: Pal from *Yersinia pestis*. Amino acid sequences were aligned using Multalin [43], and the figure was prepared using ESPript (version 3.0, http://espript.ibcp.fr/) [44].

A previous study has shown that the EAEC TagL subunit (accession numbers: EC042_4528; GI: 284924249) bears a PGBD that is required for the function of the Sci-1 T6SS. TagL is a polytopic inner membrane protein, and its PGBD locates at the periplasmic C terminus [25]. We first attempted to produce the whole periplasmic domain of TagL (amino acids 348–572). This domain exhibited limited solubility, and crystallization trials failed. The periplasmic domain comprises the C terminal PGBD *per se* (amino acids 436–554) separated from the third transmembrane segment by a linker. In order to clone the PGBD alone, we first define its boundaries. The TagL protein sequence was subjected to the secondary structure homology and prediction server HHpred [42]. HHpred returned a long list of homologous structures with HHpred probability > 99.9%. They all belong to the outer membrane protein A (OmpA) family or OmpA-like domains (e.g., bacterial flagellar motor MotB or peptidoglycan associated lipoprotein Pal). A sequence alignment with selected sequences corresponding to known structures (OmpA and Pal from *Acinetobacter baumannii*, and Pal from *Yersinia pestis*) showed that the TagL PGBD is restricted between residues Thr440 and Pro552 (Fig 1). The DNA sequence corresponding to the TagL 440–552 amino acids fragment was cloned, and the new construct was product at a high yield with good solubility. Gel filtration analyses suggested that the TagL PGBD behaves as a monomer in solution (S1 Fig).

### Crystal structure of the EAEC TagL PGBD

**Fig 2:**
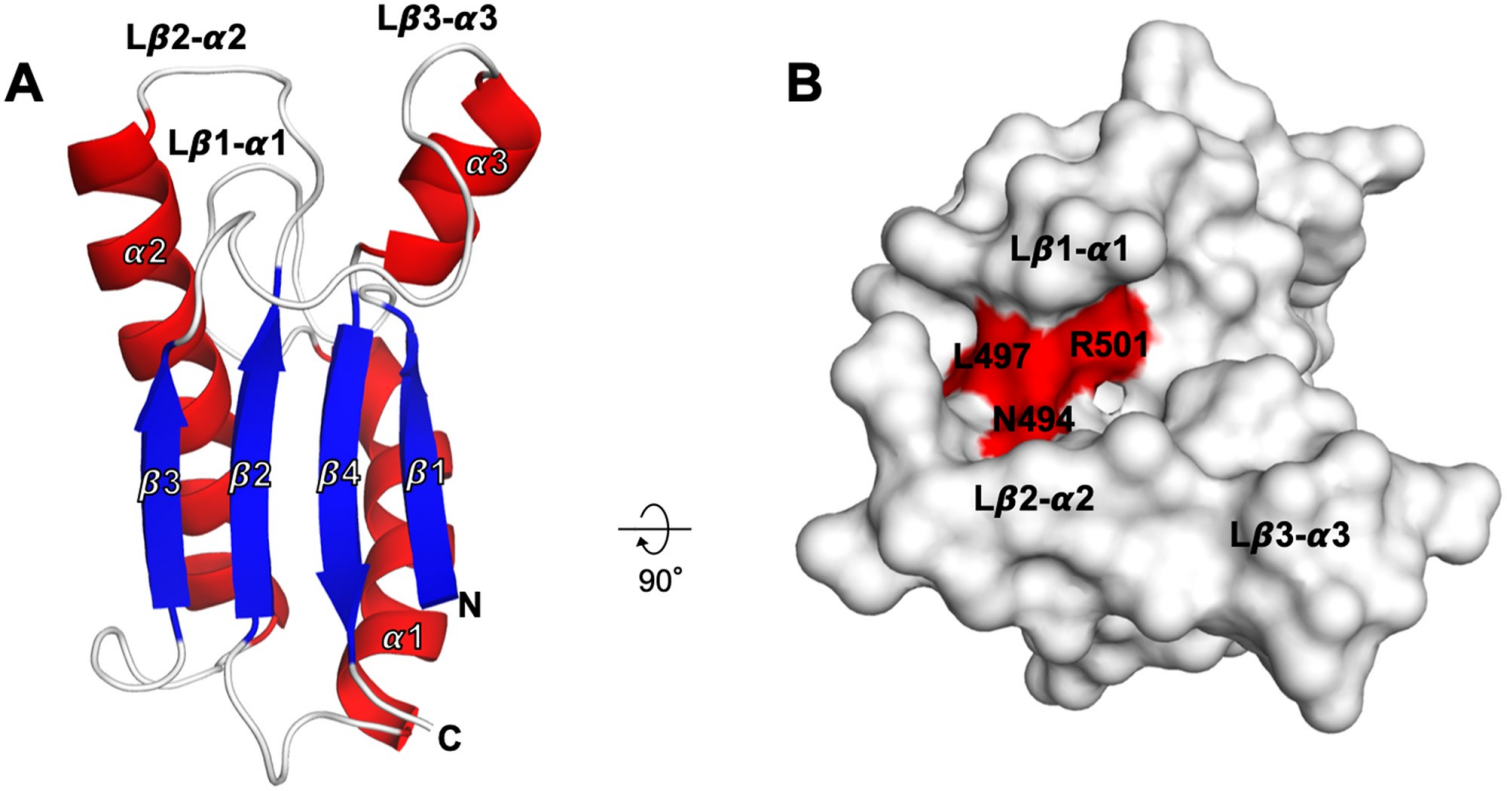
Overall structure of the TagL peptidoglycan binding domain (PGBD). A. The TagL PGBD is displayed as a ribbon diagram with blue β-sheets and red α-helices; the N and C termini are labelled. B. The surface of the TagL PGBD is displayed, with the residues involved in the cell wall binding coloured in red. The figure was prepared using PyMOL (version 1.20, https://pymol.org).

**Fig 3:**
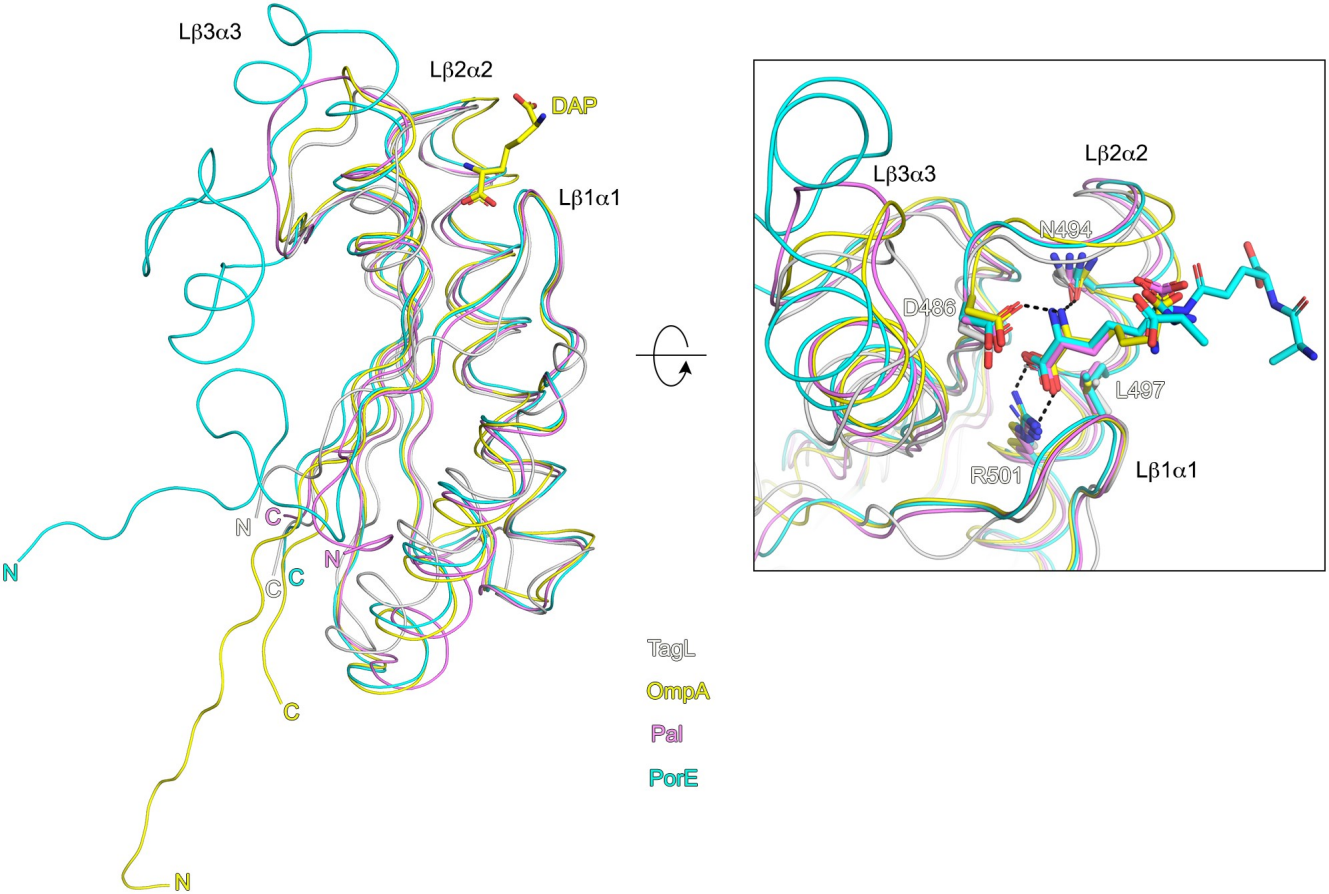
Superimposition of the TagL peptidoglycan binding domain (PGBD) with other OmpA family proteins in complex with a peptidoglycan (PG) fragment. Right: TagL PGDB, *P*. *gingivalis* PorE (6TOP), *A*. *baumannii* Pal (4G4V) and *A*. *baumannii* OmpA (3TD4) are displayed in white, cyan, pink and yellow, respectively. The N- and C-terminal extremities are labelled; for clarity purpose the structures are displayed as worm diagram, and only the DAP bound to *A*. *baumannii* OmpA is displayed, in stick format. Enclosed: Close-up view of the PG binding site. The PG fragments as well as the side chains of the conserved residues that interact with the DAP residue are displayed in stick format; hydrogen bonds are displayed as dashed lines. For clarity purpose, only the TagL residues are labelled. The figure was prepared using PyMOL (version 1.20, https://pymol.org).

Crystals of the TagL PGBD grew readily and its structure was solved by molecular refinement at 2.43Å. The structure of the TagL PGBD consists of three α-helices around a four-stranded β-sheet with connectivity β1-α1-β2-α2-β3-α3-β4, β4 being antiparallel to the other β-strands (Fig 2A). The surface of the PGBD exhibits a deep crevice defined by three loops: Lβ1-α1, Lβ2-α2 and Lβ3-α3. Among the five residues that were previously shown to be required for TagL binding to the cell wall (Asn494, Leu497, Ser498, Arg501 and Ala502 [25]), the residues Asn494, Leu497 and Arg501 locate in this crevice (Fig 2B). As expected, a DALI [45] search returned many hits of structures from the OmpA/Pal/MotB family. Superimposition with such proteins in complex with PG fragments (OmpA and Pal from *A*. *baumannii*, and PorE from *P*. *gingivalis*) strongly suggests that TagL interacts with PG. In the complex structures, the PG lies in the crevice described above, with the DAP residue buried into the molecule core between the Lβ1-α1 and Lβ2-α2 loops (Fig 3). The structures are very similar, the major difference concerning the Lβ3-α3 loop, especially in PorE from the *P*. *gingivalis* T9SS where a large insertion is present. The Lβ3-α3 loop is located quite far from the DAP burring site but could be still involved in PG binding. Its conformational diversity could therefore be related to the structural diversity of PG among bacterial species. Alternatively, the Lβ3-α3 loop could be involved in modulation of PG binding, or in interaction with protein partner(s). Nevertheless, the residues forming the DAP binding site are relatively conserved in TagL, especially the residues that interact with the DAP residue through hydrogen bonds and hydrophobic contacts: Asp486, Asn494, Leu 497 and Arg501 (Fig 3). This observation further confirms at the structural level the importance of the TagL residues Asn494, Leu497 and Arg501 whose mutation was shown to abolish interaction with the cell wall. The two other residues important for the cell wall binding (Ser498 and Ala502) are unlikely involved in DAP interaction. As they are not located at the surface of the molecule, they are probably neither involved in interaction with another part of the PG but could be rather important for the proper DAP binding site conformation.

## Discussion

In this study, we have determined the first structure of a PGBD of a protein associated with the T6SS, that of the TagL inner membrane protein of EAEC. This domain belongs to the OmpA/Pal/MotB family and was previously shown to be required for the function of the EAEC Sci-1 T6SS [25]. As expected, the structure is highly similar to other members of the family; most importantly, the PG binding pocket is conserved as the residues involved in DAP interaction are structurally conserved, which strongly suggests that TagL binds to PG through DAP interaction in the same way as other members of the OmpA/Pal/MotB family. Thus, this structure provides another example of a PGBD and illustrates that interaction with PG through the DAP residue is conserved in proteins with different cellular functions: cellular integrity through stabilization of the cell wall for OmpA or Pal, or immobilization of large assemblies such as molecular motors or secretion systems for MotB, PorE or TagL.

Co-crystallization trials of TagL PGBD with the commercially available PG fragment iE-DAP (D-γ-Glu-*meso*-DAP) did not yield any crystals. Further investigations would be necessary to characterize an interacting ligand in order to solve the structure of the TagL PGBD in complex with a PG fragment that could confirm whether the interaction network is actually conserved. Nevertheless, it was previously shown that TagL interacts with PG, and that this interaction is essential for the T6SS function [25]. In the T6SS, TagL interacts with TssL. As ten copies of TssL are present in the TssJLM membrane complex [27], we can suppose that at least as many TagL molecules associated to the complex interact with the PG, thus resulting in the tight anchoring of the TssJLM complex to the PG layer.

While the T6SS tail is a dynamic structure, the TssJLM complex was shown to be static in EAEC cells [27] and one may hypothesize that by firmly interacting with the cell wall, TagL fixes the membrane complex and prevents lateral movement in the cell envelope. In addition, binding to the PG may allow the T6SS membrane complex to tolerate the force generated during sheath contraction. However, while most T6SS gene clusters encode for a PG-binding protein, a few are lacking such a gene, and one may ask how these T6SS function. Interestingly, the T6SS is a highly mosaic system. For example, assembly of the T6SS membrane complex requires a PG-degrading enzyme that is either encoded within the T6SS gene cluster or by a housekeeping gene [32,33]. Similarly, one may imagine that PG-binding proteins such as OmpA- or Pal-like, with genes located outside the T6SS cluster, associate with T6SS lacking PGBD to anchor the membrane complex to the cell wall.

## Supporting information

S1 Fig Size exclusion chromatography analysis of the TagL peptidoglycan binding domain (PGBD). The elution volume (77.7mL) corresponds to a monomer (15.6kDa). Arrows with molecular weights indicate the elution volumes corresponding to proteins used in the calibration experiment.
(TIF)

S1 File (PDF)
