# Supplementary figures and images for: Anchoring the T6SS to the cell wall: Crystal structure of the peptidoglycan binding domain of the TagL accessory protein
Source: PLoS One. 2021 Jul 2;16(7):e0254232. doi: 10.1371/journal.pone.0254232 (PMC8253398; doi:10.1371/journal.pone.0254232)

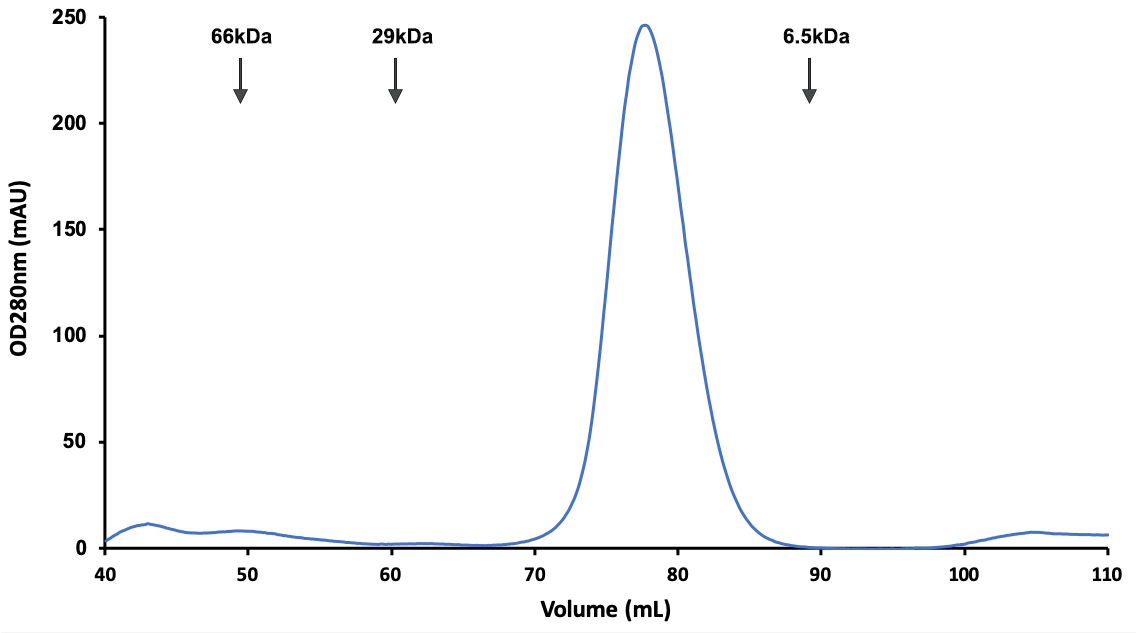

Supplement: S1 Fig — The elution volume (77.7mL) corresponds to a monomer (15.6kDa). Arrows with molecular weights indicate the elution volumes corresponding to proteins used in the calibration experiment. (TIF) [file pone.0254232.s001.tif]
