# Supplementary material for: Anchoring the T6SS to the cell wall: Crystal structure of the peptidoglycan binding domain of the TagL accessory protein
Source: PLoS One. 2021 Jul 2;16(7):e0254232. doi: 10.1371/journal.pone.0254232 (PMC8253398; doi:10.1371/journal.pone.0254232)
Supplement: S1 File — (PDF) [file pone.0254232.s002.pdf]

Full wwPDB X-ray Structure Validation Report (i)

Feb 24, 2021 – 10:41 am GMT

PDB ID	:	7BBA
Title	:	Structure of the TagL peptidoglycan binding domain from EAEC T6SS
Deposited on	:	2020-12-17
Resolution	:	2.43 Å(reported)

This is a Full wwPDB X-ray Structure Validation Report.

This report is produced by the wwPDB biocuration pipeline after annotation of the structure.

We welcome your comments at *validation@mail.wwpdb.org*
A user guide is available at
https://www.wwpdb.org/validation/2017/XrayValidationReportHelp
with specific help available everywhere you see the (i) symbol.

The following versions of software and data (see references (i)) were used in the production of this report:

MolProbity	:	4.02b-467
Mogul	:	1.8.5 (274361), CSD as541be (2020)
Xtriage (Phenix)	:	1.13
EDS	:	2.17.1.dev1
Percentile statistics	:	20191225.v01 (using entries in the PDB archive December 25th 2019)
Refmac	:	5.8.0158
CCP4	:	7.0.044 (Gargrove)
Ideal geometry (proteins)	:	Engh & Huber (2001)
Ideal geometry (DNA, RNA)	:	Parkinson et al. (1996)
Validation Pipeline (wwPDB-VP)	:	2.17.1.dev1

CONFIDENTIAL VALIDATION REPORT

1 Overall quality at a glance (i)

The following experimental techniques were used to determine the structure:
X-RAY DIFFRACTION

The reported resolution of this entry is 2.43 Å.

Percentile scores (ranging between 0-100) for global validation metrics of the entry are shown in the following graphic. The table shows the number of entries on which the scores are based.

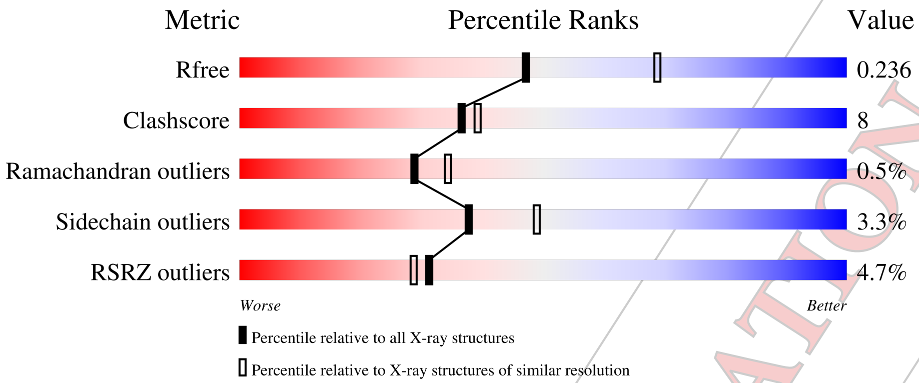

Metric	Whole archive (#Entries)	Similar resolution (#Entries, resolution range(Å))
R_{free}	130704	1564 (2.46-2.42)
Clashscore	141614	1631 (2.46-2.42)
Ramachandran outliers	138981	1617 (2.46-2.42)
Sidechain outliers	138945	1617 (2.46-2.42)
RSRZ outliers	127900	1547 (2.46-2.42)

The table below summarises the geometric issues observed across the polymeric chains and their fit to the electron density. The red, orange, yellow and green segments of the lower bar indicate the fraction of residues that contain outliers for >=3, 2, 1 and 0 types of geometric quality criteria respectively. A grey segment represents the fraction of residues that are not modelled. The numeric value for each fraction is indicated below the corresponding segment, with a dot representing fractions <=5% The upper red bar (where present) indicates the fraction of residues that have poor fit to the electron density. The numeric value is given above the bar.

Mol	Chain	Length	Quality of chain
1	A	137	2% / 69% 12% • 18%
1	B	137	2% / 73% 9% 18%
1	C	137	4% / 72% 9% •• 18%
1	D	137	7% / 67% 12% • 19%

CONFIDENTIAL VALIDATION REPORT

2 Entry composition (i)

There are 3 unique types of molecules in this entry. The entry contains 3684 atoms, of which 0 are hydrogens and 0 are deuteriums.

In the tables below, the ZeroOcc column contains the number of atoms modelled with zero occupancy, the AltConf column contains the number of residues with at least one atom in alternate conformation and the Trace column contains the number of residues modelled with at most 2 atoms.

- Molecule 1 is a protein called Putative type VI secretion protein.

Mol	Chain	Residues	Atoms					ZeroOcc	AltConf	Trace
1	A	112	Total 864	C 541	N 163	O 158	S 2	0	0	0
1	B	113	Total 871	C 545	N 164	O 160	S 2	0	0	0
1	C	113	Total 871	C 545	N 164	O 160	S 2	0	0	0
1	D	111	Total 857	C 536	N 162	O 157	S 2	0	0	0

There are 92 discrepancies between the modelled and reference sequences:

Chain	Residue	Modelled	Actual	Comment	Reference
A	416	MET	-	initiating methionine	UNP D3GUV9
A	417	GLY	-	expression tag	UNP D3GUV9
A	418	HIS	-	expression tag	UNP D3GUV9
A	419	HIS	-	expression tag	UNP D3GUV9
A	420	HIS	-	expression tag	UNP D3GUV9
A	421	HIS	-	expression tag	UNP D3GUV9
A	422	HIS	-	expression tag	UNP D3GUV9
A	423	HIS	-	expression tag	UNP D3GUV9
A	424	SER	-	expression tag	UNP D3GUV9
A	425	SER	-	expression tag	UNP D3GUV9
A	426	GLY	-	expression tag	UNP D3GUV9
A	427	VAL	-	expression tag	UNP D3GUV9
A	428	ASP	-	expression tag	UNP D3GUV9
A	429	LEU	-	expression tag	UNP D3GUV9
A	430	GLY	-	expression tag	UNP D3GUV9
A	431	THR	-	expression tag	UNP D3GUV9
A	432	GLU	-	expression tag	UNP D3GUV9
A	433	ASN	-	expression tag	UNP D3GUV9
A	434	LEU	-	expression tag	UNP D3GUV9
A	435	TYR	-	expression tag	UNP D3GUV9
A	436	PHE	-	expression tag	UNP D3GUV9

Continued on next page...

CONFIDENTIAL VALIDATION REPORT

Continued from previous page...

Chain	Residue	Modelled	Actual	Comment	Reference
A	437	GLN	-	expression tag	UNP D3GUV9
A	438	SER	-	expression tag	UNP D3GUV9
B	416	MET	-	initiating methionine	UNP D3GUV9
B	417	GLY	-	expression tag	UNP D3GUV9
B	418	HIS	-	expression tag	UNP D3GUV9
B	419	HIS	-	expression tag	UNP D3GUV9
B	420	HIS	-	expression tag	UNP D3GUV9
B	421	HIS	-	expression tag	UNP D3GUV9
B	422	HIS	-	expression tag	UNP D3GUV9
B	423	HIS	-	expression tag	UNP D3GUV9
B	424	SER	-	expression tag	UNP D3GUV9
B	425	SER	-	expression tag	UNP D3GUV9
B	426	GLY	-	expression tag	UNP D3GUV9
B	427	VAL	-	expression tag	UNP D3GUV9
B	428	ASP	-	expression tag	UNP D3GUV9
B	429	LEU	-	expression tag	UNP D3GUV9
B	430	GLY	-	expression tag	UNP D3GUV9
B	431	THR	-	expression tag	UNP D3GUV9
B	432	GLU	-	expression tag	UNP D3GUV9
B	433	ASN	-	expression tag	UNP D3GUV9
B	434	LEU	-	expression tag	UNP D3GUV9
B	435	TYR	-	expression tag	UNP D3GUV9
B	436	PHE	-	expression tag	UNP D3GUV9
B	437	GLN	-	expression tag	UNP D3GUV9
B	438	SER	-	expression tag	UNP D3GUV9
C	416	MET	-	initiating methionine	UNP D3GUV9
C	417	GLY	-	expression tag	UNP D3GUV9
C	418	HIS	-	expression tag	UNP D3GUV9
C	419	HIS	-	expression tag	UNP D3GUV9
C	420	HIS	-	expression tag	UNP D3GUV9
C	421	HIS	-	expression tag	UNP D3GUV9
C	422	HIS	-	expression tag	UNP D3GUV9
C	423	HIS	-	expression tag	UNP D3GUV9
C	424	SER	-	expression tag	UNP D3GUV9
C	425	SER	-	expression tag	UNP D3GUV9
C	426	GLY	-	expression tag	UNP D3GUV9
C	427	VAL	-	expression tag	UNP D3GUV9
C	428	ASP	-	expression tag	UNP D3GUV9
C	429	LEU	-	expression tag	UNP D3GUV9
C	430	GLY	-	expression tag	UNP D3GUV9
C	431	THR	-	expression tag	UNP D3GUV9
C	432	GLU	-	expression tag	UNP D3GUV9

Continued on next page...

CONFIDENTIAL VALIDATION REPORT

Continued from previous page...

Chain	Residue	Modelled	Actual	Comment	Reference
C	433	ASN	-	expression tag	UNP D3GUV9
C	434	LEU	-	expression tag	UNP D3GUV9
C	435	TYR	-	expression tag	UNP D3GUV9
C	436	PHE	-	expression tag	UNP D3GUV9
C	437	GLN	-	expression tag	UNP D3GUV9
C	438	SER	-	expression tag	UNP D3GUV9
D	416	MET	-	initiating methionine	UNP D3GUV9
D	417	GLY	-	expression tag	UNP D3GUV9
D	418	HIS	-	expression tag	UNP D3GUV9
D	419	HIS	-	expression tag	UNP D3GUV9
D	420	HIS	-	expression tag	UNP D3GUV9
D	421	HIS	-	expression tag	UNP D3GUV9
D	422	HIS	-	expression tag	UNP D3GUV9
D	423	HIS	-	expression tag	UNP D3GUV9
D	424	SER	-	expression tag	UNP D3GUV9
D	425	SER	-	expression tag	UNP D3GUV9
D	426	GLY	-	expression tag	UNP D3GUV9
D	427	VAL	-	expression tag	UNP D3GUV9
D	428	ASP	-	expression tag	UNP D3GUV9
D	429	LEU	-	expression tag	UNP D3GUV9
D	430	GLY	-	expression tag	UNP D3GUV9
D	431	THR	-	expression tag	UNP D3GUV9
D	432	GLU	-	expression tag	UNP D3GUV9
D	433	ASN	-	expression tag	UNP D3GUV9
D	434	LEU	-	expression tag	UNP D3GUV9
D	435	TYR	-	expression tag	UNP D3GUV9
D	436	PHE	-	expression tag	UNP D3GUV9
D	437	GLN	-	expression tag	UNP D3GUV9
D	438	SER	-	expression tag	UNP D3GUV9

- Molecule 2 is SULFATE ION (three-letter code: SO4) (formula: O_4S).

CONFIDENTIAL VALIDATION REPORT

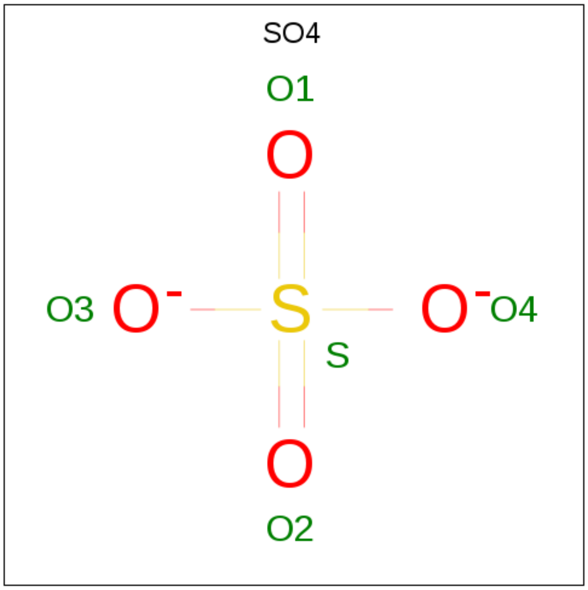

Mol	Chain	Residues	Atoms	ZeroOcc	AltConf
2	A	1	Total O S<br>5 4 1	0	0
2	A	1	Total O S<br>5 4 1	0	0
2	B	1	Total O S<br>5 4 1	0	0
2	B	1	Total O S<br>5 4 1	0	0
2	C	1	Total O S<br>5 4 1	0	0
2	C	1	Total O S<br>5 4 1	0	0
2	C	1	Total O S<br>5 4 1	0	0
2	D	1	Total O S<br>5 4 1	0	0
2	D	1	Total O S<br>5 4 1	0	0

- Molecule 3 is water.

Mol	Chain	Residues	Atoms	ZeroOcc	AltConf
3	A	63	Total O<br>63 63	0	0
3	B	56	Total O<br>56 56	0	0
3	C	26	Total O<br>26 26	0	0

Continued on next page...

CONFIDENTIAL VALIDATION REPORT

Continued from previous page...

Mol	Chain	Residues	Atoms	ZeroOcc	AltConf
3	D	31	Total O<br>31 31	0	0

CONFIDENTIAL VALIDATION REPORT

3 Residue-property plots

These plots are drawn for all protein, RNA, DNA and oligosaccharide chains in the entry. The first graphic for a chain summarises the proportions of the various outlier classes displayed in the second graphic. The second graphic shows the sequence view annotated by issues in geometry and electron density. Residues are color-coded according to the number of geometric quality criteria for which they contain at least one outlier: green = 0, yellow = 1, orange = 2 and red = 3 or more. A red dot above a residue indicates a poor fit to the electron density (RSRZ > 2). Stretches of 2 or more consecutive residues without any outlier are shown as a green connector. Residues present in the sample, but not in the model, are shown in grey.

- Molecule 1: Putative type VI secretion protein

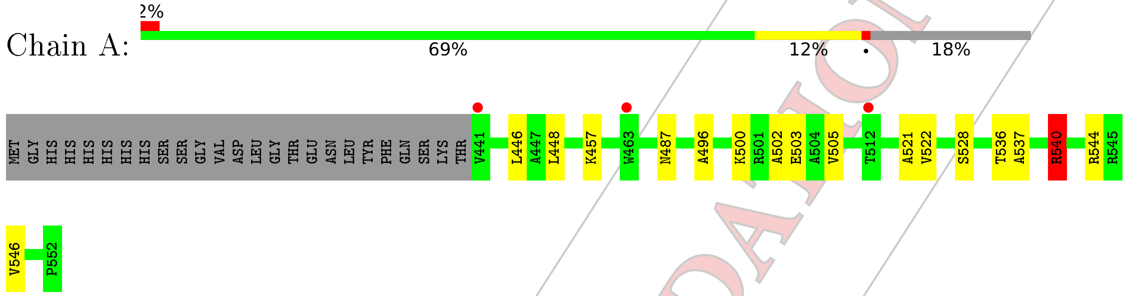

- Molecule 1: Putative type VI secretion protein

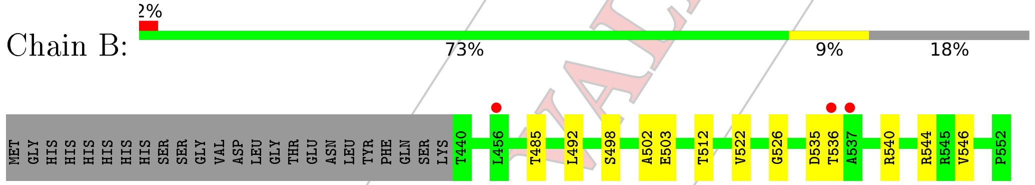

- Molecule 1: Putative type VI secretion protein

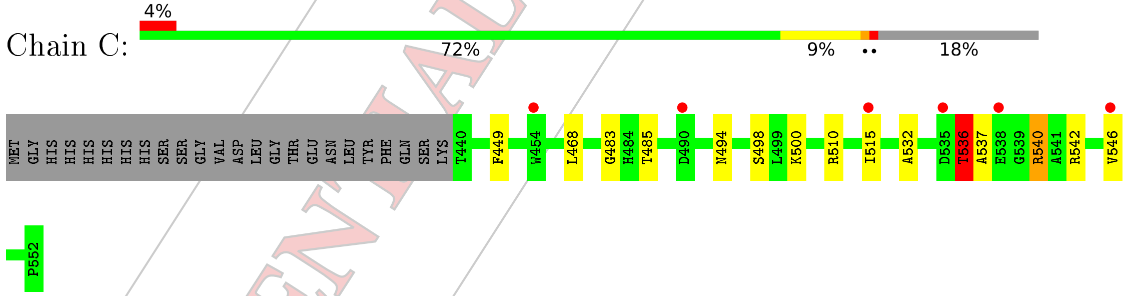

- Molecule 1: Putative type VI secretion protein

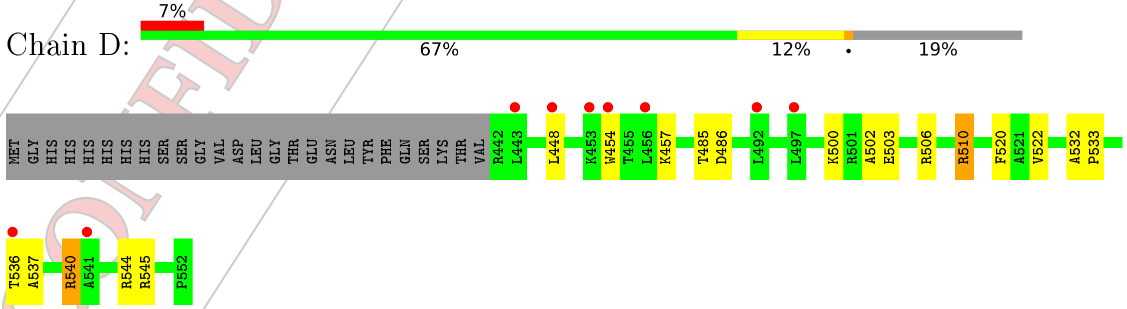

CONFIDENTIAL VALIDATION REPORT

4 Data and refinement statistics (i)

Property	Value	Source
Space group	P 63	Depositor
Cell constants<br>a, b, c, α, β, γ	75.89Å 75.89Å 177.67Å<br>90.00° 90.00° 120.00°	Depositor
Resolution (Å)	34.90 – 2.43<br>34.90 – 2.43	Depositor<br>EDS
% Data completeness<br>(in resolution range)	100.0 (34.90-2.43)<br>100.0 (34.90-2.43)	Depositor<br>EDS
R_{merge}	(Not available)	Depositor
R_{sym}	(Not available)	Depositor
$< I/\sigma(I) >$ [1]	1.64 (at 2.42Å)	Xtriage
Refinement program	BUSTER 2.10.4 (11-DEC-2020)	Depositor
R, R_{free}	0.237 , 0.246<br>0.227 , 0.236	Depositor<br>DCC
R_{free} test set	1089 reflections (5.00%)	wwPDB-VP
Wilson B-factor (Å^2)	58.8	Xtriage
Anisotropy	0.355	Xtriage
Bulk solvent k_{sol}(e/Å^3), B_{sol}(Å^2)	0.32 , 52.4	EDS
L-test for twinning[2]	$< \|L\| > = 0.43$, $< L^2 > = 0.26$	Xtriage
Estimated twinning fraction	0.267 for h,-h-k,-l	Xtriage
F_o,F_c correlation	0.94	EDS
Total number of atoms	3684	wwPDB-VP
Average B, all atoms (Å^2)	75.0	wwPDB-VP

Xtriage's analysis on translational NCS is as follows: *The largest off-origin peak in the Patterson function is 4.45% of the height of the origin peak. No significant pseudotranslation is detected.*

[1] Intensities estimated from amplitudes.
[2] Theoretical values of $< |L| >$, $< L^2 >$ for acentric reflections are 0.5, 0.333 respectively for untwinned datasets, and 0.375, 0.2 for perfectly twinned datasets.

CONFIDENTIAL VALIDATION REPORT

5 Model quality (i)

5.1 Standard geometry (i)

Bond lengths and bond angles in the following residue types are not validated in this section: SO4

The Z score for a bond length (or angle) is the number of standard deviations the observed value is removed from the expected value. A bond length (or angle) with $|Z| > 5$ is considered an outlier worth inspection. RMSZ is the root-mean-square of all Z scores of the bond lengths (or angles).

Mol	Chain	Bond lengths		Bond angles	
		RMSZ	$\#\|Z\| > 5$	RMSZ	$\#\|Z\| > 5$
1	A	0.48	0/881	0.67	0/1197
1	B	0.45	0/888	0.64	0/1207
1	C	0.43	0/888	0.64	0/1207
1	D	0.42	0/874	0.66	0/1187
All	All	0.45	0/3531	0.65	0/4798

Chiral center outliers are detected by calculating the chiral volume of a chiral center and verifying if the center is modelled as a planar moiety or with the opposite hand.A planarity outlier is detected by checking planarity of atoms in a peptide group, atoms in a mainchain group or atoms of a sidechain that are expected to be planar.

Mol	Chain	#Chirality outliers	#Planarity outliers
1	A	0	1
1	D	0	1
All	All	0	2

There are no bond length outliers.

There are no bond angle outliers.

There are no chirality outliers.

All (2) planarity outliers are listed below:

Mol	Chain	Res	Type	Group
1	A	540	ARG	Sidechain
1	D	510	ARG	Sidechain

CONFIDENTIAL VALIDATION REPORT

5.2 Too-close contacts (i)

In the following table, the Non-H and H(model) columns list the number of non-hydrogen atoms and hydrogen atoms in the chain respectively. The H(added) column lists the number of hydrogen atoms added and optimized by MolProbity. The Clashes column lists the number of clashes within the asymmetric unit, whereas Symm-Clashes lists symmetry-related clashes.

Mol	Chain	Non-H	H(model)	H(added)	Clashes	Symm-Clashes
1	A	864	0	872	18	0
1	B	871	0	879	12	0
1	C	871	0	879	12	0
1	D	857	0	863	14	0
2	A	10	0	0	0	0
2	B	10	0	0	0	0
2	C	15	0	0	0	0
2	D	10	0	0	0	0
3	A	63	0	0	1	0
3	B	56	0	0	0	0
3	C	26	0	0	0	0
3	D	31	0	0	0	0
All	All	3684	0	3493	55	0

The all-atom clashscore is defined as the number of clashes found per 1000 atoms (including hydrogen atoms). The all-atom clashscore for this structure is 8.

All (55) close contacts within the same asymmetric unit are listed below, sorted by their clash magnitude.

Atom-1	Atom-2	Interatomic distance (Å)	Clash overlap (Å)
1:A:505:VAL:HG21	1:A:546:VAL:HG11	1.61	0.83
1:C:449:PHE:CE2	1:C:546:VAL:HG23	2.17	0.80
1:D:502:ALA:CB	1:D:522:VAL:HG13	2.13	0.78
1:C:449:PHE:HE2	1:C:546:VAL:HG23	1.51	0.75
1:C:536:THR:HA	1:C:540:ARG:HB2	1.72	0.72
1:A:540:ARG:HG3	1:A:544:ARG:HH12	1.55	0.71
1:B:536:THR:HG23	1:B:540:ARG:HH11	1.56	0.70
1:A:536:THR:HA	1:A:540:ARG:CD	2.23	0.69
1:D:502:ALA:HB1	1:D:522:VAL:HG13	1.76	0.67
1:C:536:THR:HG23	1:C:537:ALA:H	1.58	0.67
1:D:502:ALA:HB3	1:D:522:VAL:HG13	1.77	0.65
1:A:446:LEU:HB2	1:A:546:VAL:HG12	1.79	0.64
1:B:536:THR:HA	1:B:540:ARG:HD3	1.80	0.63
1:C:536:THR:HG23	1:C:537:ALA:N	2.15	0.62
1:A:446:LEU:HB2	1:A:546:VAL:CG1	2.29	0.62

Continued on next page...

CONFIDENTIAL VALIDATION REPORT

Continued from previous page...

Atom-1	Atom-2	Interatomic distance (Å)	Clash overlap (Å)
1:C:532:ALA:HB2	1:C:542:ARG:HD2	1.82	0.60
1:A:502:ALA:CB	1:A:522:VAL:HG13	2.32	0.60
1:A:540:ARG:HG3	1:A:544:ARG:NH1	2.17	0.58
1:D:503:GLU:HG3	1:D:522:VAL:HG11	1.85	0.58
1:A:536:THR:HA	1:A:540:ARG:HD3	1.84	0.57
1:D:536:THR:HA	1:D:540:ARG:HB3	1.88	0.56
1:C:485:THR:HG21	1:C:494:ASN:O	2.06	0.55
1:A:536:THR:HA	1:A:540:ARG:HD2	1.89	0.53
1:B:485:THR:HG23	1:B:498:SER:HB3	1.90	0.52
1:B:502:ALA:CB	1:B:522:VAL:HG13	2.39	0.52
1:A:502:ALA:HB3	1:A:522:VAL:HG13	1.92	0.51
1:C:536:THR:CG2	1:C:537:ALA:N	2.74	0.51
1:A:503:GLU:HG3	1:A:522:VAL:HG11	1.92	0.50
1:B:503:GLU:HG3	1:B:522:VAL:HG11	1.92	0.50
1:A:502:ALA:HB1	1:A:522:VAL:HG13	1.94	0.50
1:B:512:THR:HG22	1:B:512:THR:O	2.11	0.50
1:D:485:THR:HG22	1:D:486:ASP:H	1.77	0.49
1:C:485:THR:OG1	1:C:494:ASN:HB3	2.13	0.48
1:A:521:ALA:HA	1:B:522:VAL:O	2.13	0.48
1:C:485:THR:HG23	1:C:498:SER:HB3	1.95	0.48
1:D:540:ARG:HE	1:D:544:ARG:HH12	1.60	0.48
1:A:536:THR:O	1:A:537:ALA:HB3	2.14	0.46
1:B:540:ARG:HE	1:B:544:ARG:HH12	1.62	0.46
1:D:502:ALA:HB3	1:D:522:VAL:CG1	2.46	0.45
1:B:502:ALA:HB1	1:B:522:VAL:HG13	1.99	0.45
1:B:536:THR:HG23	1:B:540:ARG:NH1	2.27	0.44
1:D:536:THR:HB	1:D:540:ARG:HD2	1.97	0.44
1:D:454:TRP:CE2	1:D:500:LYS:HE3	2.53	0.43
1:B:485:THR:OG1	1:B:526:GLY:C	2.58	0.43
1:B:502:ALA:HB3	1:B:522:VAL:HG13	2.00	0.42
1:C:483:GLY:HA3	1:C:498:SER:O	2.20	0.42
1:D:532:ALA:O	1:D:533:PRO:C	2.57	0.42
1:A:528:SER:O	3:A:701:HOH:O	2.21	0.42
1:A:496:ALA:O	1:A:500:LYS:HG2	2.20	0.41
1:C:468:LEU:CD1	1:C:515:ILE:HD11	2.50	0.41
1:D:506:ARG:HG3	1:D:520:PHE:CG	2.56	0.41
1:A:500:LYS:HD2	1:A:503:GLU:OE1	2.21	0.40
1:D:448:LEU:O	1:D:457:LYS:HB2	2.20	0.40
1:D:486:ASP:OD2	1:D:540:ARG:HB2	2.20	0.40
1:A:448:LEU:O	1:A:457:LYS:HB2	2.21	0.40

There are no symmetry-related clashes.

CONFIDENTIAL VALIDATION REPORT

5.3 Torsion angles (i)

5.3.1 Protein backbone (i)

In the following table, the Percentiles column shows the percent Ramachandran outliers of the chain as a percentile score with respect to all X-ray entries followed by that with respect to entries of similar resolution.

The Analysed column shows the number of residues for which the backbone conformation was analysed, and the total number of residues.

Mol	Chain	Analysed	Favoured	Allowed	Outliers	Percentiles	
1	A	110/137 (80%)	105 (96%)	5 (4%)	0	100	100
1	B	111/137 (81%)	106 (96%)	5 (4%)	0	100	100
1	C	111/137 (81%)	105 (95%)	5 (4%)	1 (1%)	17	20
1	D	109/137 (80%)	104 (95%)	4 (4%)	1 (1%)	17	20
All	All	441/548 (80%)	420 (95%)	19 (4%)	2 (0%)	29	34

All (2) Ramachandran outliers are listed below:

Mol	Chain	Res	Type
1	D	537	ALA
1	C	536	THR

5.3.2 Protein sidechains (i)

In the following table, the Percentiles column shows the percent sidechain outliers of the chain as a percentile score with respect to all X-ray entries followed by that with respect to entries of similar resolution.

The Analysed column shows the number of residues for which the sidechain conformation was analysed, and the total number of residues.

Mol	Chain	Analysed	Rotameric	Outliers	Percentiles	
1	A	90/112 (80%)	88 (98%)	2 (2%)	52	64
1	B	91/112 (81%)	88 (97%)	3 (3%)	38	49
1	C	91/112 (81%)	87 (96%)	4 (4%)	28	37
1	D	89/112 (80%)	86 (97%)	3 (3%)	37	48
All	All	361/448 (81%)	349 (97%)	12 (3%)	38	49

All (12) residues with a non-rotameric sidechain are listed below:

CONFIDENTIAL VALIDATION REPORT

Mol	Chain	Res	Type
1	A	487	ASN
1	A	540	ARG
1	B	492	LEU
1	B	535	ASP
1	B	546	VAL
1	C	500	LYS
1	C	510	ARG
1	C	536	THR
1	C	540	ARG
1	D	510	ARG
1	D	540	ARG
1	D	545	ARG

Sometimes sidechains can be flipped to improve hydrogen bonding and reduce clashes. There are no such sidechains identified.

5.3.3 RNA (i)

There are no RNA molecules in this entry.

5.4 Non-standard residues in protein, DNA, RNA chains (i)

There are no non-standard protein/DNA/RNA residues in this entry.

5.5 Carbohydrates (i)

There are no monosaccharides in this entry.

5.6 Ligand geometry (i)

9 ligands are modelled in this entry.

In the following table, the Counts columns list the number of bonds (or angles) for which Mogul statistics could be retrieved, the number of bonds (or angles) that are observed in the model and the number of bonds (or angles) that are defined in the Chemical Component Dictionary. The Link column lists molecule types, if any, to which the group is linked. The Z score for a bond length (or angle) is the number of standard deviations the observed value is removed from the expected value. A bond length (or angle) with $|Z| > 2$ is considered an outlier worth inspection. RMSZ is the root-mean-square of all Z scores of the bond lengths (or angles).

CONFIDENTIAL VALIDATION REPORT

Mol	Type	Chain	Res	Link	Bond lengths			Bond angles		
					Counts	RMSZ	#|Z| > 2	Counts	RMSZ	#|Z| > 2
2	SO4	D	602	-	4,4,4	0.82	0	6,6,6	1.38	1 (16%)
2	SO4	A	602	-	4,4,4	0.14	0	6,6,6	0.23	0
2	SO4	B	602	-	4,4,4	0.30	0	6,6,6	0.23	0
2	SO4	C	603	-	4,4,4	0.22	0	6,6,6	0.29	0
2	SO4	B	601	-	4,4,4	0.13	0	6,6,6	0.15	0
2	SO4	D	601	-	4,4,4	0.15	0	6,6,6	0.15	0
2	SO4	C	602	-	4,4,4	0.23	0	6,6,6	0.25	0
2	SO4	C	601	-	4,4,4	0.19	0	6,6,6	0.30	0
2	SO4	A	601	-	4,4,4	0.26	0	6,6,6	0.22	0

There are no bond length outliers.

All (1) bond angle outliers are listed below:

Mol	Chain	Res	Type	Atoms	Z	Observed(^{o})	Ideal(^{o})
2	D	602	SO4	O4-S-O2	3.08	125.38	109.31

There are no chirality outliers.

There are no torsion outliers.

There are no ring outliers.

No monomer is involved in short contacts.

5.7 Other polymers (i)

There are no such residues in this entry.

5.8 Polymer linkage issues (i)

There are no chain breaks in this entry.

CONFIDENTIAL VALIDATION REPORT

6 Fit of model and data (i)

6.1 Protein, DNA and RNA chains (i)

In the following table, the column labelled '#RSRZ> 2' contains the number (and percentage) of RSRZ outliers, followed by percent RSRZ outliers for the chain as percentile scores relative to all X-ray entries and entries of similar resolution. The OWAB column contains the minimum, median, 95th percentile and maximum values of the occupancy-weighted average B-factor per residue. The column labelled 'Q< 0.9' lists the number of (and percentage) of residues with an average occupancy less than 0.9.

Mol	Chain	Analysed	<RSRZ>	#RSRZ>2			OWAB(Å^2)	Q<0.9
1	A	112/137 (81%)	0.25	3 (2%)	54	50	43, 64, 83, 97	0
1	B	113/137 (82%)	0.25	3 (2%)	54	50	45, 66, 87, 100	0
1	C	113/137 (82%)	0.47	6 (5%)	26	23	62, 79, 108, 111	0
1	D	111/137 (81%)	0.66	9 (8%)	12	9	60, 89, 109, 113	0
All	All	449/548 (81%)	0.41	21 (4%)	31	29	43, 74, 104, 113	0

All (21) RSRZ outliers are listed below:

Mol	Chain	Res	Type	RSRZ
1	C	538	GLU	4.1
1	C	454	TRP	3.9
1	D	536	THR	3.7
1	D	443	LEU	3.6
1	D	456	LEU	3.4
1	B	537	ALA	2.9
1	D	492	LEU	2.9
1	D	448	LEU	2.9
1	D	454	TRP	2.8
1	C	490	ASP	2.7
1	A	441	VAL	2.5
1	B	536	THR	2.5
1	D	541	ALA	2.4
1	C	535	ASP	2.4
1	D	453	LYS	2.2
1	C	515	ILE	2.1
1	B	456	LEU	2.1
1	A	463	TRP	2.1
1	D	497	LEU	2.1
1	C	546	VAL	2.0
1	A	512	THR	2.0

CONFIDENTIAL VALIDATION REPORT

6.2 Non-standard residues in protein, DNA, RNA chains (i)

There are no non-standard protein/DNA/RNA residues in this entry.

6.3 Carbohydrates (i)

There are no monosaccharides in this entry.

6.4 Ligands (i)

In the following table, the Atoms column lists the number of modelled atoms in the group and the number defined in the chemical component dictionary. The B-factors column lists the minimum, median, 95th percentile and maximum values of B factors of atoms in the group. The column labelled 'Q< 0.9' lists the number of atoms with occupancy less than 0.9.

Mol	Type	Chain	Res	Atoms	RSCC	RSR	B-factors($Å^2$)	Q<0.9
2	SO4	D	602	5/5	0.69	0.20	116,116,116,116	5
2	SO4	C	601	5/5	0.92	0.17	92,92,92,92	0
2	SO4	C	602	5/5	0.92	0.29	72,72,72,73	5
2	SO4	B	601	5/5	0.92	0.15	98,98,98,98	0
2	SO4	C	603	5/5	0.94	0.20	78,78,78,79	5
2	SO4	D	601	5/5	0.94	0.13	102,102,102,102	0
2	SO4	B	602	5/5	0.94	0.18	85,85,85,86	0
2	SO4	A	602	5/5	0.95	0.17	64,64,64,64	5
2	SO4	A	601	5/5	0.97	0.09	73,73,73,74	0

6.5 Other polymers (i)

There are no such residues in this entry.

CONFIDENTIAL VALIDATION REPORT
